# Characterization of neuraminidase inhibitor-resistant influenza virus isolates from immunocompromised patients in the Republic of Korea

**DOI:** 10.1186/s12985-020-01375-1

**Published:** 2020-07-06

**Authors:** Heui Man Kim, Namjoo Lee, Mi-Seon Kim, Chun Kang, Yoon-Seok Chung

**Affiliations:** Division of Viral Diseases, Center for Laboratory Control of Infectious Diseases, Korea Centers for Disease Control and Prevention, Cheongju-si, South Korea

**Keywords:** Influenza virus, Drug resistance, H275Y, Immunocompromised patients

## Abstract

**Background:**

The emergence of influenza viruses resistant to anti-influenza drugs is a threat to global public health. The Korea Centers for Disease Control and Prevention operates the Korea Influenza and Respiratory Viruses Surveillance System (KINRESS) to monitor epidemics of influenza and Severe Acute Respiratory Infection (SARI) to identify mutated influenza viruses affecting drug resistance, pathogenesis, and transmission.

**Methods:**

Oropharyngeal swab samples were collected from KINRESS and SARI during the 2018–2019 season. The specimens confirmed influenza virus using real-time RT-PCR on inoculated MDCK cells. HA and NA sequences of the influenza viruses were analyzed for phylogeny and mutations. Neuraminidase inhibition and hemagglutination inhibition assays were utilized to characterize the isolates.

**Results:**

Two A(H1N1)pdm09 isolates harboring an H275Y substitution in the neuraminidase sequence were detected in patients with acute hematologic cancer. They had prolonged respiratory symptoms, with the virus present in the respiratory tract despite oseltamivir and peramivir treatment. Through the neuraminidase inhibition assay, both viruses were found to be resistant to oseltamivir and peramivir, but not to zanamivir. Although hemagglutinin and neuraminidase phylogenetic analyses suggested that the 2 A(H1N1)pdm09 isolates were not identical, their antigenicity was similar to that of the 2018–19 influenza vaccine virus.

**Conclusions:**

Our data indicate the utility of monitoring influenza-infected immunocompromised patients in general hospitals for the early detection of emerging neuraminidase inhibitor-resistant viruses and maintaining continuous laboratory surveillance of patients with influenza-like illness in sentinel clinics to monitor the spread of such new variants. Finally, characterization of the virus can inform the risk assessment for future epidemics and pandemics caused by drug-resistant influenza viruses.

## Background

Neuraminidase inhibitors (NAIs) are globally utilized for the treatment and prevention of influenza types A and B infection [1], which are stockpiled by some countries, including the Republic of Korea, against unexpected pandemics. NAIs suppress the action of neuraminidase (NA) on the surface of the virus to prevent the spread of progeny virus from infected cells [2]. Oseltamivir and peramivir, the most commonly used drugs for patients with influenza-like illnesses in the Republic of Korea, help to relieve clinical symptoms within 2 days of symptom manifestation and shorten the virus-release period in the respiratory tract [1]. Baloxavir, a cap-dependent endonuclease inhibitor, was also recently licensed in the United States, following Japan [3]. However, since most recently detected type A influenza viruses harbor the resistance variation S31N in the matrix gene, the M2 proton-channel blockers amantadine and rimantadine are no longer clinically applied [4].

The Korea Centers for Diseases Control and Prevention (KCDC) has operated the Korea Influenza and Respiratory Viruses Surveillance System (KINRESS) to monitor epidemic features of influenza viruses and analyze virus characteristics, including drug resistance, since 2000. The KCDC carries out diagnostic testing for influenza viruses in respiratory specimens requested by general hospitals as a national standard laboratory. If a drug resistance mutation is found in the NA gene, the KINRESS attempts to isolate the virus and perform phenotypic analysis (NA inhibition assay). The typical drug-resistance substitutions in NA include H275Y, E119D/G, and Q136R for A(H1N1)pdm09; E119V, D151G/V/D, R224K, E276D, R292K, and N294S for A (H3N2); and G104E, E117A/D, H134Y, and R150K for B virus [5], although additional single and combination mutations may also result in NAI drug resistance (World Health Organization (WHO)) [6]. Flusurver provides genetic analysis tools for screening drug-resistance and variant mutations to facilitate genetic characterization of viruses [7]. Fluorescence-based NA inhibition assays using the MUNANA substrate have been conducted to confirm drug-resistant virus phenotyping [8]. NAI-resistant viruses were identified in cases in which influenza virus was continuously detected following NAI treatment of hospitalized, immunosuppressed patients, rather than in clinical outpatients [9]. Here, drug-resistant A(H1N1)pdm09 viruses were detected via the KINRESS in patients with acute hematologic cancer not exhibiting recovery despite oseltamivir and peramivir administration; these were characterized genetically and antigenically following isolation.

## Methods

### Clinical specimen collection

Oropharyngeal swab samples were collected from patients with influenza-like illness through the KINRESS and processed in the national standard laboratory. Swabs were stored in Viral Transport Media (BD, San Jose, CA, USA) until further analysis.

### Viral RNA extraction

Viral RNA was extracted from 140 μL of sample medium using the QIAamp Viral RNA Mini Kit (Qiagen, Hilden, Germany), according to the manufacturer’s instructions.

### One-step multiplex real-time reverse transcription-polymerase chain reaction (RT-PCR)

RT-PCR was carried out using the Agpath ID One-step RT-PCR system (Applied Biosystems, Foster City, CA, USA) [10]. Table 1 lists multiplex (A/B/IPC and H1/H3/IPC) primer and Taq-Man probe sets. The 20-μL reaction mixture contained 5 μL of RNA template, 590 nM primers, 140 nM probe, 10 μL of 2× reaction buffer, 0.8 μL of enzyme mixture, and RNase-free water. RT-PCR was performed on the ABI 7500Fast instrument (Applied Biosystems) with thermocycler conditions for reverse transcription (50 °C, 30 min), hot start DNA Taq polymerase activation (95 °C, 10 min), followed by 40 cycles of denaturation (95 °C, 15 s) and annealing/extension (55 °C, 30 s). Data acquisition and analysis of the real-time PCR assay were performed using SDS software Version 1.4 (Applied Biosystems).

**Table 1:** Primer and probe sequence information to detect influenza virus type (A/B) and subtype (H1/H3)

Type/Subtype	Primer/probe	Sequence (5′–3′)	Target (bp)
A	Forward	AATCCTGTCACCTCTGACTAAGG	Matrix (98)
	Reverse	CATTYTGGACAAAKCGTCTACG	
	Probe	FAM-TGCAGTCCTCGCTCAC-MGBNFQ	
B	Forward	GAATGCTGTCAATGAATATTGAGGG	Nucleoprotein (77)
	Reverse	CATTGAGTCATTCATCATCTTGAGTAGAT	
	Probe	VIC-TCCTTTGACATCTGCAT-MGBNFQ	
H1	Forward	AACAATTCAACAGACACTGTAGACACAGT	Hemagglutinin (144)
	Reverse	GGGCTACCCCTCTTAGTTTRCATAGTTT	
	Probe	FAM- ATGTAACAGTAACACACTCTGT-MGBNFQ	
H3	Forward	GGAATGGTTTGTCATTGGGAAT	Hemagglutinin (95)
	Reverse	AAGCTCAATAATGAGRTCAGATGCA	
	Probe	VIC-CTTCCATTTGGAGTRATRCA-MGBNFQ	
IPC	Forward	AGATTTGGACCTGCGAGCG	*GAPDH* (65)
	Reverse	GAGCGGCTGTCTCCACAAGT	
	Probe	Cy5-TTCTGACCTGAAGGCTCTGCGCG-BHQ	

### Virus isolation in MDCK cell lines

Madin-Darby canine kidney (MDCK, American Type Culture Collection, Manassas, VA, USA) cells were seeded at 10^5^/mL in T25 flasks with Dulbecco’s modified Eagle medium (Hyclone, Logan, UT, USA) containing 10% fetal bovine serum (Hyclone) and 1% penicillin–streptomycin (Sigma, St. Louis, MO, USA) in a final volume of 5 mL and incubated at 37 °C in a humidified atmosphere with 5% CO_2_ for 48 h. When the cells reached 90% confluence, the culture medium was discarded, and the cells were washed three times with warm phosphate-buffered saline. Then, 0.2 mL of clinical specimen was added into a flask to allow the inoculum to adsorb (37 °C, 60 min). Medium (5 mL) containing 2 μL/mL TPCK-trypsin was added to the flask. The culture was observed daily for cytopathic and morphological changes using an inverted light microscope. The culture supernatant was harvested when 75–100% of the infected cells showed cytopathic effect and stored at − 70 °C [11]. Finally, A/Korea/S0002/2019 and A/Korea/S0003/2019 A(H1N1)pdm09 viruses were isolated.

### Hemagglutinin (HA) and neuraminidase (NA) sequencing

Viral RNA was extracted from oropharyngeal swab samples and their isolates using the QIAamp Viral RNA Mini Kit (Qiagen) according to the manufacturer’s instructions. Reverse transcription was performed to obtain cDNA using the U12 primer, then the HA and NA genes were amplified with specific primers [12]. The PCR products were purified using the QIAquick™ PCR Purification Kit (Qiagen) according to the manufacturer’s instructions and subjected to direct sequencing using the Big Dye Terminator V.3.0 Cycle Sequencing Ready Reaction Kit (Applied Biosystems) together with primers for each PCR fragment (Table 2) on the ABI 3130xl Genetic Analyzer automatic sequencer (Applied Biosystems). The nucleotide sequences were edited, assembled, and aligned using MEGA X 10.1 software (https://www.megasoftware.net/). The complete HA (GISAID: EPI1602907 and EPI1602905) and NA (GISAID: EPI1602908 and EPI1602906) sequences were obtained from the 2 A(H1N1)pdm09 viruses.

**Table 2:** Sequencing primers for HA and NA of A(H1N1)pdm09 virus

Primers (F/R)	Gene (subtype)	Locus	Sequence (5′–3′)
A-uni-HA-1F	HA	1–28	TATTCGTCTCAGGGAGCAAAAGCAGGGG
HA-H1-345F	HA (H1)	345–365	GGAACGTGTTACCCAGGAGAT
HA-H1-680F	HA (H1)	680–701	TCAAGCCGGAAATAGCAATAAG
HA-H1-1137F	HA (H1)	1137–1156	AGCCGACCTGAAGAGCACAC
HA-H1-714R	HA (H1)	691–714	CCTCACTTTGGGTCTTATTGCTAT
HA-H1-1093R	HA (H1)	1072–1093	CATCCATCTACCATCCCTGTCC
HA-H1-1330R	HA (H1)	1309–1330	GCATTGTAAGTCCAAATGTCCA
A-uni-HA-1807R	HA	1773–1807	ATATCGTCTCGTATTAGTAGAAACAAGGGTGTTTT
A-uni-NA-1F	NA	1–29	TATTGGTCTCAGGGAGCAAAAGCAGGAGT
H1-NA-324F	NA (N1)	324–345	GGTTCCAAGGGGGATGTGTTTG
H1-NA-744F	NA (N1)	744–766	TGGACAGGCCTCATACAAGATCT
H1-NA-645R	NA (N1)	622–645	AGTGTCTGTTATTATTCCGTTGTA
H1-NA-1010F	NA (N1)	985–1010	CAGTATCGTCTAATGGAGCAAATGGA
H1-NA-1057R	NA (N1)	1036–1057	CTATCCAAACACCATTGCCGTA
A-uni-NA-1442R	NA	1407–1442	ATATGGTCTCGTATTAGTAGAAACAAGGAGTTTTTT

### Genetic characterization

Genetic screening for drug resistance in the NA gene and mutational analysis of the HA and NA genes were conducted using Flusurver (https://flusurver.bii.a-star.edu.sg/), last updated on July 26, 2019. Phylogenies were reconstructed using the neighbor-joining method and bootstrapped 1000 times with MEGA X 10.1 using HA- or NA-aligned DNA sequences of the A(H1N1)pdm09 reference vaccine [13] and other isolates from the Republic of Korea.

### NA inhibition assay

A fluorescence-based neuraminidase inhibition assay was conducted using the NA-Fluor™ Influenza Neuraminidase Assay Kit (Applied Biosystems), according to the manufacturer’s instructions. The susceptibility of influenza viruses to NAI was characterized using oseltamivir, zanamivir, and peramivir at concentrations that inhibited the NA activity by 50% (IC_50_) as described previously [14]. As defined by the WHO, influenza A viruses with normal, reduced, and highly reduced NA activity inhibition exhibit a < 10-fold, 10- to 100-fold, and ≥ 100-fold increase in IC_50_, respectively; the last are considered clinically resistant [15].

### Hemagglutination inhibition (HI) assay

Assays were performed according to standard methods [16]. Four HA units/25 μL of A(H1N1)pdm09 virus were tested using 0.5% suspensions of turkey red blood cells. HI titers were reciprocals of the highest dilutions of sera that inhibited hemagglutination. Post-infection ferret antisera against 18–19 and 19–20 vaccine viruses were treated with receptor-destroying enzyme (Denka Seiken, Tokyo, Japan).

## Results

### A(H1N1)pdm09 detection and isolation in immunocompromised patients

Influenza A virus was detected in oropharyngeal swab samples collected from patients with lymphoblastic leukemia (Patient A: female, 37 years old) and relapsed lymphoma (Patient B: female, 38 years old), with real-time RT-PCR Ct values of 28 and 29, respectively. A(H1N1)pdm09 viruses were identified by additional H1 subtype detection at respective Ct values of 28 and 29 (Table 3). Plaques were observed within 48 h following specimen inoculation in MDCK cells, with the harvested supernatant exhibiting an HA titer of 256. The isolates were named A/Korea/S0002/2019 and A/Korea/S0003/2019.

**Table 3:** A(H1N1)pdm09 virus detection in immunocompromised patients by real-time RT-PCR

Patient	Genetic detection using real-time RT-PCR				
	IFV A	IFV B	A/H1	A/H3	IPC
A	26.1	UD^*^	25.4	UD^*^	26.3/26.2
B	27.0	UD^*^	25.8	UD^*^	32.1/34.0

UD^*^: undetected

### Genetic characterization

#### NA genetic analysis for screening drug resistance, phylogeny, and variation

**Fig. 1:**
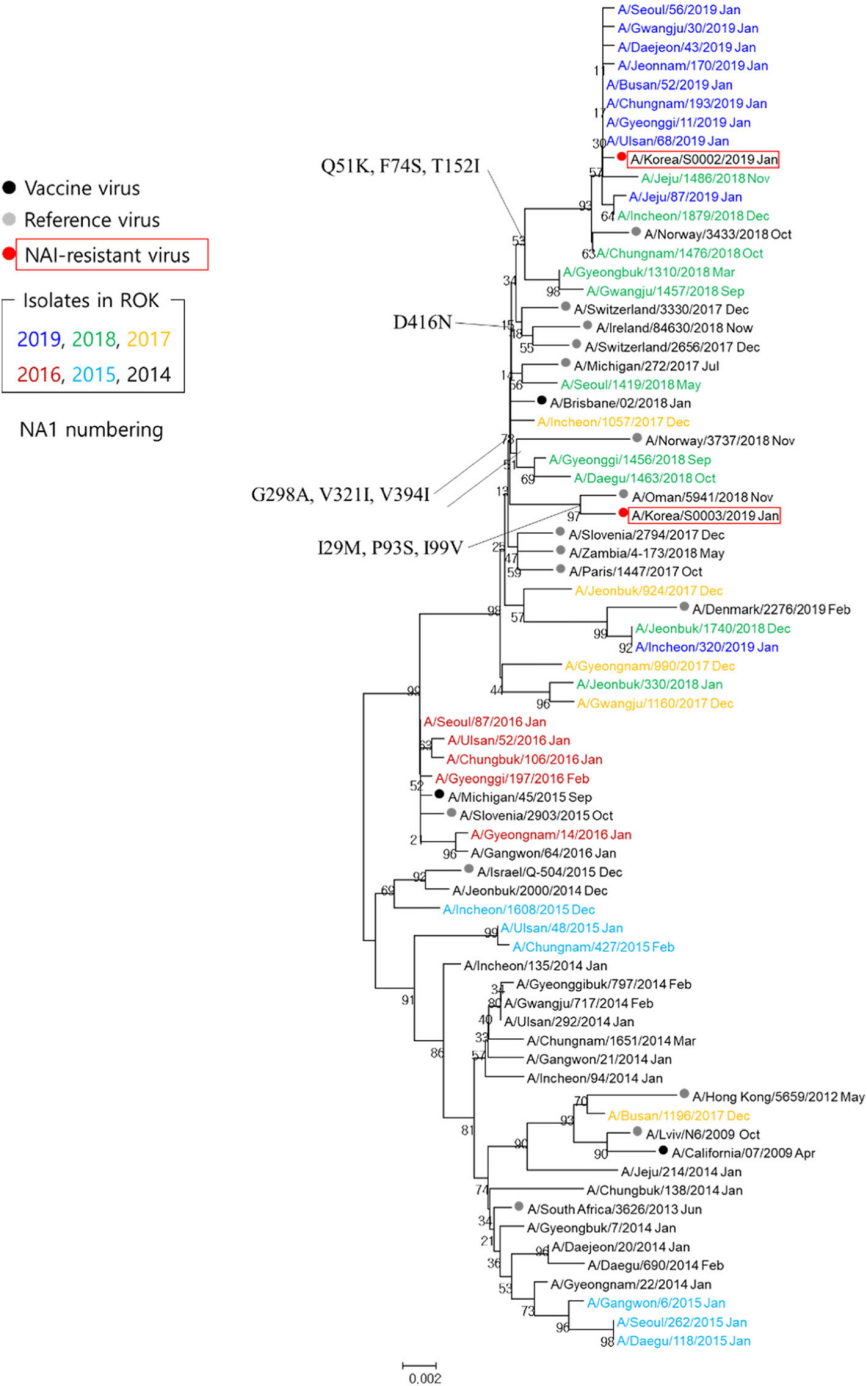
Phylogenetic tree of neuraminidase (NA) gene of drug resistant A(H1N1)pdm09. The H275Y mutated viruses (A/Korea/S0002/2019 and A/Korea/S0003/2019) were not identical in phylogenetic tree of NA. A/Korea/S0002/2019 harbored Q51K, F74S, T152I and D416N mutation and A/Korea/S0003/2019 harbored I29M, P93S, I99V, G298A, V321I and V394I mutation compared to A/Brisbane/02/2018

The sequences of the NA gene from oropharyngeal swab samples and the isolated viruses from each patient were determined and were found to have no mutations resulting from adaptation to the cell culture conditions. The H275Y NA substitution (N1 numbering), associated with strong drug resistance, was observed in (GISAID: EPI1602908 and EPI1602906) of A(H1N1)pdm09 isolates from both immunocompromised patients. Other NA drug-resistance substitutions (V116A, I117V, Q136K, D151A, Y155H, R156K, D198V, I222V, R224K, Q226H, E227D, E277Q, R293K, N294S, E425G, and I436N) were not detected. The NA sequences of A/Korea/S0002/2019 and A/Korea/S0003/2019 most closely matched with those of A/Brisbane/02/2018 (98.5 and 98.3% amino-acid identity, respectively). The viruses could be distinguished in the phylogenetic tree (Fig. 1). A/Korea/S0002/2019 had six amino acid differences (T13I, Q51K, F74S, H275Y, D416N, and T452I) relative to A/Brisbane/02/2018 (Table 4), while A/Korea/S0003/2019 had eight (T13I, I29M, P93S, I99V, H275Y, G298A, V321I, and V394I). Table 4 shows the amino acid substitutions in NA compared with A/Brisbane/02/2018 and their reported effects.

**Table 4:** NA genetic mutations compared with A/Brisbane/02/2018 (N1 numbering)

Virus	Amino acid identity with A/Brisbane/02/2018 (%)	T13I	I29M	Q51K	F74S	P93S^a,b^	I99V^b^	H275Y^**c**^	G298A^a^	V321I^a^	V394I^b^	D416N^a,b^	T452I^a,b,d^
A/Korea/S0003/2019	98.3	○	○			○	○	○	○	○	○		
A/Korea/S0002/2019	98.5	○		○	○			○				○	○
Frequency (%)		99.79	0.65	18.14	16.78	0.34	0.89	0.58	0.75	1.13	1.37	36.25	18.76

○ substitution of the amino acid indicated

^a^Binding small ligand

^b^viral oligomerization interfaces

^c^strong drug resistance

^d^binding host protein

#### HA phylogeny and variation

**Fig. 2:**
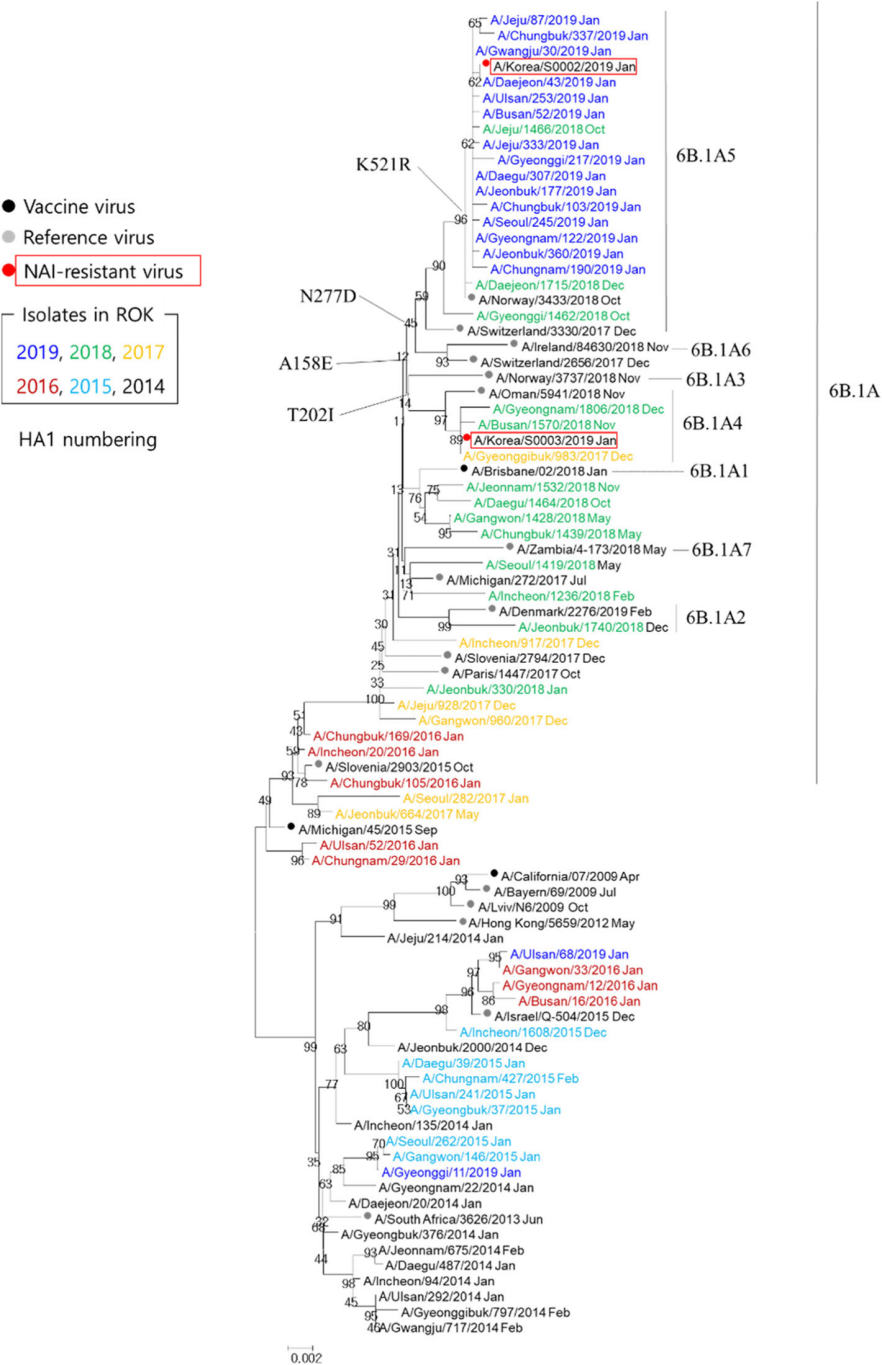
Phylogenetic tree of hemagglutinin (HA) gene of drug resistant A(H1N1)pdm09. A/Korea/S0002/2019 virus belonged to 6B.1A5 harboring T202I, N277D and K521R mutations compared to A/Brisbane/02/2018 and clustered with isolated viruses in 2019 year, Republic of Korea (ROK), whereas A/Korea/S0003/2019 virus belonged to 6B.1A4 harboring A158E mutation and clustered with isolated viruses in previous year

In the HA phylogenetic tree, two viruses belonged to clade 6B.1A, including A/Brisbane/02/2018 and a virus similar to a 2018–2019 season-isolated A(H1N1)pdm09 in the Republic of Korea. A/Korea/S0002/2019 and A/Korea/S0003/2019 HA sequences best matched with those of A/Brisbane/02/2018 (98.6 and 98.9% amino-acid identity, respectively). The two viruses fell into different subclades according to discrepancies at residues 158, 202, 277, and 521. A/Korea/S0002/2019 belonged to subclade 6B.1A5 and A/Korea/S0003/2019 to subclade 6B.1A4 (Fig. 2). Table 5 shows the amino-acid substitutions in the HA gene compared with A/Brisbane/02/2018 and their reported effects.

**Table 5:** HA genetic variation compared with A/Brisbane/02/2018 (H1 numbering)

Virus	Amino acid identity with A/Brisbane/02/2018 (%)	G62R^a,b,c^	N146D^a,b,c^	A158E ^a,b,c^	T202I ^a,b,c^	R240Q ^a,c,d^	N277D^b^	A299P^a,b^	V315I^a,b^	K521^b^
A/Korea/S0003/2019	98.94	○	○	○		○		○	○	
A/Korea/S0002/2019	98.59	○	○		○	○	○	○	○	○
Frequency (%)		84.19	17.76	0.97	19.77	99.87	29.46	84.85	85.38	3.08

○ substitution of the amino acid indicated

^a^Binding small ligand

^b^viral oligomerization interfaces

^c^antibody recognition sites

^d^host cell receptor binding

### NA inhibition assay

A/Korea/S0003/2019 and A/Korea/S0002/2019 viruses exhibited significantly reduced inhibition by oseltamivir and peramivir with > 100 IC_50_ fold-changes relative to drug-sensitive virus, but were normally susceptible to zanamivir (Table 6). However, every isolate collected from the KINRESS in 2018–2019 was susceptible to all three drugs.

**Table 6:** Neuraminidase inhibition assay for A(H1N1)pdm09 viruses isolated in immunocompromised patients

Virus	Type (Subtype)	Genotype	NAI resistance (IC_50_ fold change)		
			Oseltamivir	Zanamivir	Peramivir
A/Korea/S0003/2019	A(H1N1)pdm09	Variant (H275Y)	HRI 179.33	NI 2.56	HRI 117.74
A/Korea/S0002/2019		Variant (H275Y)	HRI 221.58	NI 0.44	HRI 168.48
2018–2019 isolates	A(H1N1)pdm09	Wild	0.1–1.2	0.3–0.7	0.1
	A(H3N2)	Wild	0.1–0.2	0.4–0.8	0.2–0.3
	B	Wild	9–16	0.7–1.6	0.3–0.4

### Antigenic characterization

The two viruses were well inhibited by antisera from ferrets immunized with A/Michigan/45/2015 and A/Brisbane/02/2018, which are northern-hemisphere influenza A(H1N1)pdm09 isolates collected in 2018–2019 and 2019–2020, respectively, with less than two-fold reduced HI titer relative to homologous virus (Table 7).

**Table 7:** Hemagglutinin inhibition assay

Virus	Hemagglutination inhibition titer	
	Post-infection ferret antisera	
	A/Michigan/45/2015 Egg	A/Brisbane/02/2018 Egg
A/Korea/S0003/2019 MDCK	640	640
A/Korea/S0002/2019 MDCK	320	640
A/Michigan/45/2015 MDCK	1280	640
A/Brisbane/02/2018 MDCK	640	1280

## Discussion

The NAI-resistant A(H1N1) virus harboring the NA gene H275Y variation was first identified in 2007, and was frequently identified worldwide in 2008–2009 [17]. However, since the 2009 A(H1N1)pdm09 pandemic, it occurred in < 1% of A(H1N1)pdm09, mainly reported in patients with impaired immune systems due to immunosuppressive therapies for conditions including cancer and organ transplants [18, 19]. Notably, such immunocompromised patients, when infected with influenza virus, could shed virus over a prolonged period, likely causing increased transmission. Moreover, as they shed drug-resistant virus while maintaining transmissibility, these patients may constitute a source for spreading drug-resistant influenza in the community [18].

Although NAI-resistant virus has been rarely observed since 2000, the KCDC, through the KINRESS, identified oseltamivir and peramivir-resistant A(H1N1)pdm09 viruses harboring the H275Y NA variation in patients with acute lymphoblastic leukemia and relapsed lymphoma hospitalized in the same general hospital, with both exhibiting prolonged virus excretion. Although there was no history of the two patients occupying the same ward, we were concerned about potential NAI-resistant virus transmission. The phylogenetic analysis showed that the two isolated A(H1N1)pdm09 viruses were genetically distinct. Moreover, there were no additional reports of similar viruses in that hospital. Furthermore, van der Vries et al. [20] demonstrated that A(H1N1)pdm09 virus-infected immunocompromised ferrets exhibited prolonged virus replication despite antiviral therapy, along with the H275Y substitution observed in the virus population from day 8 onwards only in ferrets that received oseltamivir. These results suggest that the H275Y substitution emerges rapidly in immunocompromised hosts under continuous antiviral selective pressure.

Mutations suspected to represent antigenic drift in immunocompromised patients were more likely to be observed in NA than HA, albeit at a very low frequency (around 1%). However, the NA mutations did not affect antigenic properties, besides drug resistance. Although the HA N158D substitution increases the binding affinity of H5N1 avian influenza virus and human receptor (α2–6-linked sialoside) [21], the function of the A/Korea/S0002/2019 HA A158E substitution functionality remains unknown (but it was not involved in antigenicity in this study). Alternatively, NA and HA had different mutation spectra in swine influenza viruses collected from April to June 2019 in the United States. The antigenic drift in immunocompromised patients may allow swine influenza virus insertion to occur without pig to human transmission, ultimately reducing the efficacy of influenza vaccine escape. Nevertheless, antisera from ferrets immunized with the influenza vaccine exhibited good inhibition of A/Korea/S0002/2019 and A/Korea/S0003/2019 infection.

## Conclusions

The NA H275Y substitution and some HA or NA substitutions in drug-resistant A(H1N1)pdm09 viruses isolated from immunocompromised patients influence both antigenicity and NAI resistance. The viruses showed highly reduced inhibition, with over 100-fold increases in the IC_50_ for oseltamivir and peramivir, but not zanamivir. Thus, zanamivir might be an option for immunocompromised patients when oseltamivir and peramivir are not effective. These findings also indicate the necessity of monitoring for NAI-resistant viruses in outpatients visiting clinics and in-patients, particularly for immunocompromised patients (e.g., with severe acute respiratory syndromes in general hospitals). Additionally, virus characterization can facilitate the risk assessment of NAI viruses.

## Data Availability

The data supporting the conclusions of this article are included within the article.

## Abbreviations

KINRESS: Korea Influenza and Respiratory Viruses Surveillance System
SARI: Severe Acute Respiratory Infection
H: Histidine
Y: Tyrosine
NAIs: Neuraminidase inhibitors
NA: Neuraminidase
HA: Hemagglutinin
MDCK: Madin-Darby canine kidney
GISAID: Global Initiative on Sharing All Influenza Data
IC_50_: Half maximal inhibitory concentration
